# Successful Management of a Chronic Pedal Ulcer Secondary to Snake Bite: A Case Report

**DOI:** 10.7759/cureus.104325

**Published:** 2026-02-26

**Authors:** Joseph Jose, Saji Jose, Lakshmi Prakash, Dimmy Harold

**Affiliations:** 1 Cardiovascular Medicine, Cardiff Health Centre, Manarcad, IND; 2 Internal Medicine, Cardiff Health Centre, Manarcad, IND; 3 Plastic and Reconstructive Surgery, Caritas Matha Hospital, Kottayam, IND

**Keywords:** anti-inflammatory drugs, chronic inflammation, chronic non-healing ulcer, snake bite, wound care techniques

## Abstract

Chronic ulceration following snake-bite envenoming is a severely disabling and therapeutic challenge, often refractory to multiple treatment modalities. We report the successful management of a non-healing pedal ulcer, 30 years in duration, on the left lower limb of a 53-year-old man, secondary to a viper bite. The ulcer had a three-decade history of recurrence despite various treatments, including a failed surgical skin graft 15 years earlier. Upon presentation, extensive workup ruled out vascular insufficiency, infection, osteomyelitis, and malignancy. A wound biopsy confirmed chronic inflammation, leading to a diagnosis of a chronic inflammatory ulcer. Management focused on suppressing the underlying inflammatory drive. The patient was started on a course of anti-inflammatory therapy with deflazacort and colchicine, alongside standard wound care and pain control. Over eight months, the inflammation subsided, and healthy granulation tissue formed. While maintaining anti-inflammatory cover, a split-thickness skin graft was performed, which was entirely successful. The patient was successfully weaned off medications and remains ulcer-free at one-year follow-up. This case highlights that the key to managing such longstanding, refractory ulcers lies in recognizing and targeting the persistent chronic inflammation over an extended period before attempting surgical reconstruction. This novel strategy of prolonged medical therapy to create a conducive wound environment was critical to the success of the skin graft, in which all previous interventions had failed, offering a potential new paradigm for managing this challenging sequela of snake bite.

## Introduction

Snakes are a significant stimulus that causes fear and sometimes even phobias in humans. It is hypothesized that this fear arose initially from predators like pythons. Later, as venomous snakes evolved, mortality from their defensive bites also became an important factor [1]. Snake-bite envenoming constitutes a significant neglected tropical disease globally, with an estimated 1.2-5.5 million cases annually, resulting in over 125,000 fatalities and permanent disability or disfigurement in three to four times as many individuals [2]. While acute complications like tissue necrosis, coagulopathy, and renal failure are well-documented, the long-term sequelae of envenoming, particularly chronic ulceration, remain understudied and pose substantial therapeutic challenges [3]. These ulcers are frequently refractory to standard wound care and can even progress into squamous cell carcinoma [3,4]. Management strategies are largely empirical, lacking robust evidence, especially in cases that persist for decades. This report details the successful management of a recalcitrant, three-decade-old viper bite-induced leg ulcer, using anti-inflammatory therapy followed by split-thickness skin grafting, and highlights the critical role of controlling chronic inflammation prior to surgical intervention.

## Case presentation

A 53-year-old gentleman without other comorbidities came to our outpatient department with a chronic ulcer on the left lower limb, along with pain, pruritus, erythema, and purulent discharge. Detailed history revealed an incident of a viper bite 30 years ago, resulting in subsequent skin changes at the bite site. Following the event, there was an acute onset of edema associated with pain, tenderness, and erythema, which later progressed to ulceration with purulent discharge. He subsequently underwent fasciotomy, which temporarily resolved his symptoms. Two years later, there was a recurrence of ulcer at the same site, for which the patient sought Ayurvedic therapy. This only provided mild pain relief. Twenty-six years ago, he had the appearance of blisters associated with pruritus, causing abrasions, eventually leading to ulceration. This was treated with oral antibiotics along with conservative wound care. Persistence of symptoms prompted the patient to undergo a surgical skin graft 15 years ago. However, the graft was rejected within two weeks. Unfortunately, information regarding this surgical procedure was unavailable since it occurred at a different hospital, and the patient had no previous relevant medical records.

**Figure 1:**
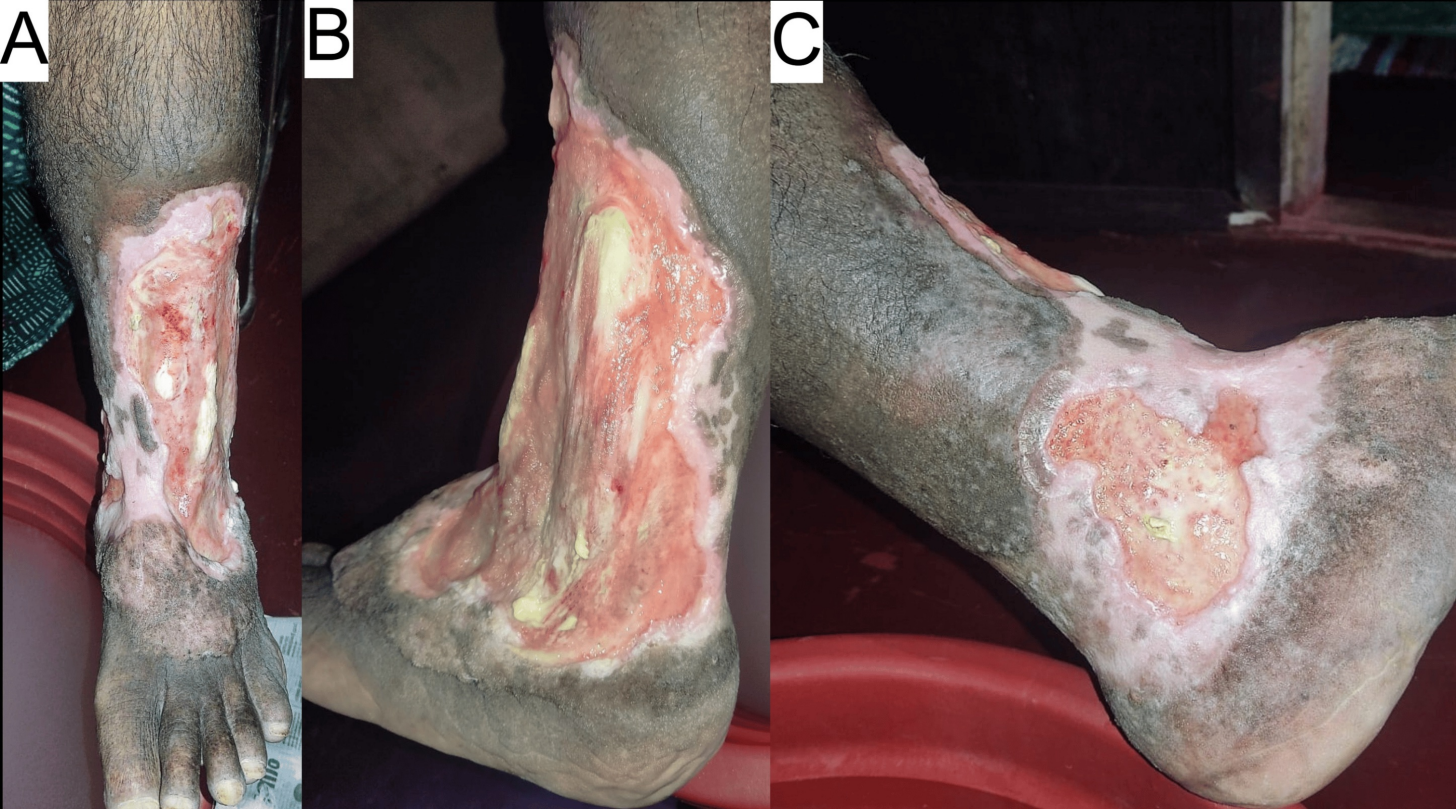
Pictures of ulcer at the time of initial presentation (patient-taken photographs) (A-C).

Upon presentation at our institution, a local examination revealed a large, irregularly shaped ulcer, mainly on the lateral aspect of the left leg, with some extension to the medial side, as shown in Figures 1A-1C. Blood tests revealed an elevated ESR of 45 mm/h, suggesting chronic inflammation. Renal function tests, full blood count, random blood glucose, and HbA1c were within normal ranges. Table 1 shows the lab work-up values.

**Table 1:** Laboratory investigations at initial presentation. RBS: random blood sugar

Blood investigations	Values	Normal reference range
Hemoglobin (g/dL)	11.8	12-18
White blood cell count (×10^9^/L)	9.1	4-10
Platelets (×10^9^/L)	321	150-500
RBS (mg/dL)	114	<140
Creatinine (mg/dL)	0.9	0.6-1.2
Urea (mg/dL)	7.2	7-20
HbA1c (%)	5.6	4.5-6.4
ESR (mm/h)	45	<15

**Figure 2:**
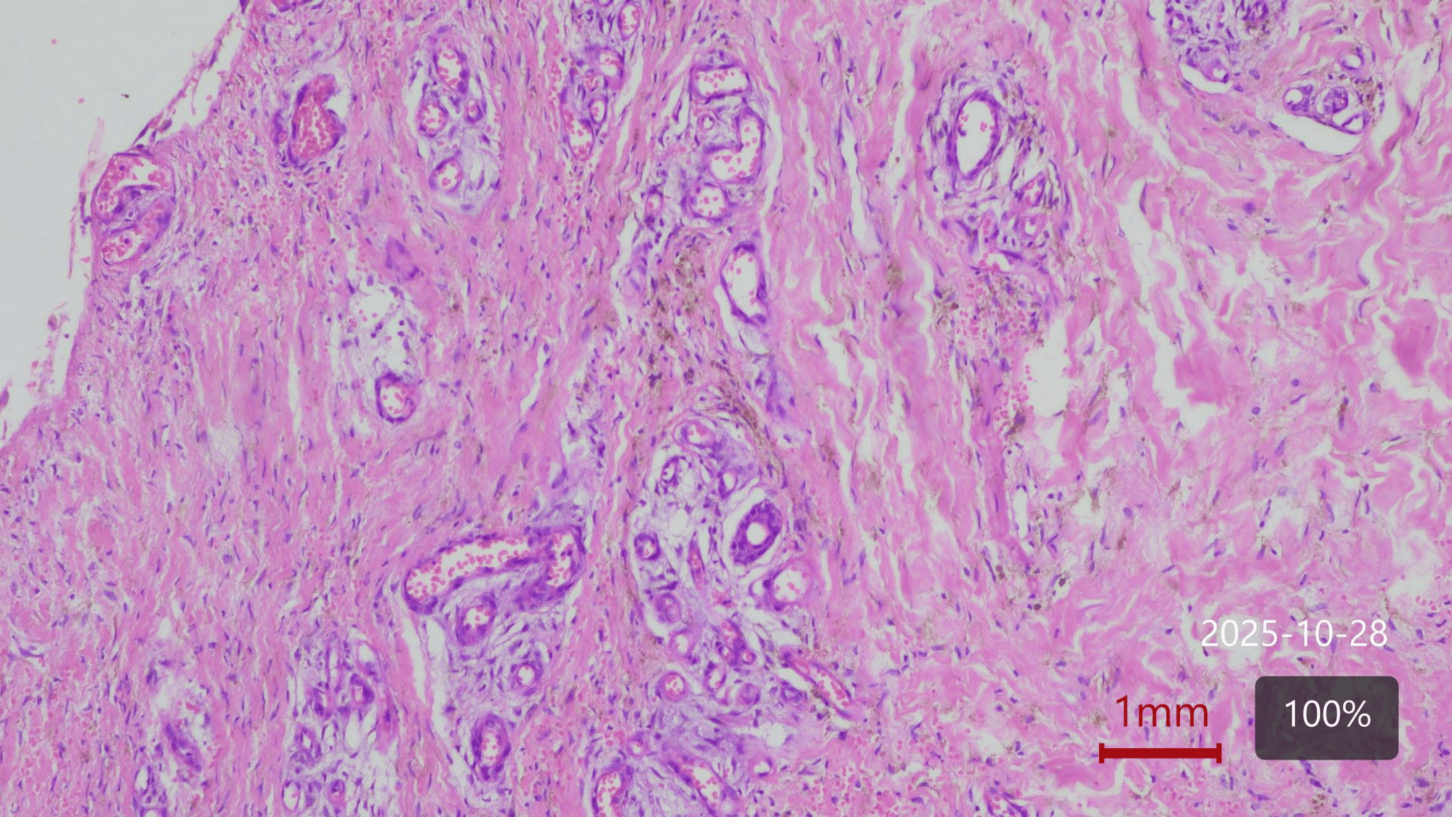
Punch biopsy of wound edge. Two skin specimens measuring 0.6x0.4x0.4 cm. The image predominantly shows features of fibrosis, peri-vascular lymphocytic infiltrate with histiocytes, extravasated erythrocytes, and hemosiderin-laden macrophages. No evidence of malignancy was seen.

Venous and arterial Doppler ultrasound scan showed no significant insufficiencies. A plain film radiograph of the left leg and foot was taken, which did not show any findings suggestive of osteomyelitis. A wound biopsy showed features of chronic inflammation and effectively ruled out malignancy. Figure 2 shows the biopsy picture. It was decided to treat this ulcer as atypical, likely of vasculitic or autoimmune etiology, based on clinical judgment and the absence of features of alternative etiologies, such as vascular, malignant, or chronic infections.

**Figure 3:**
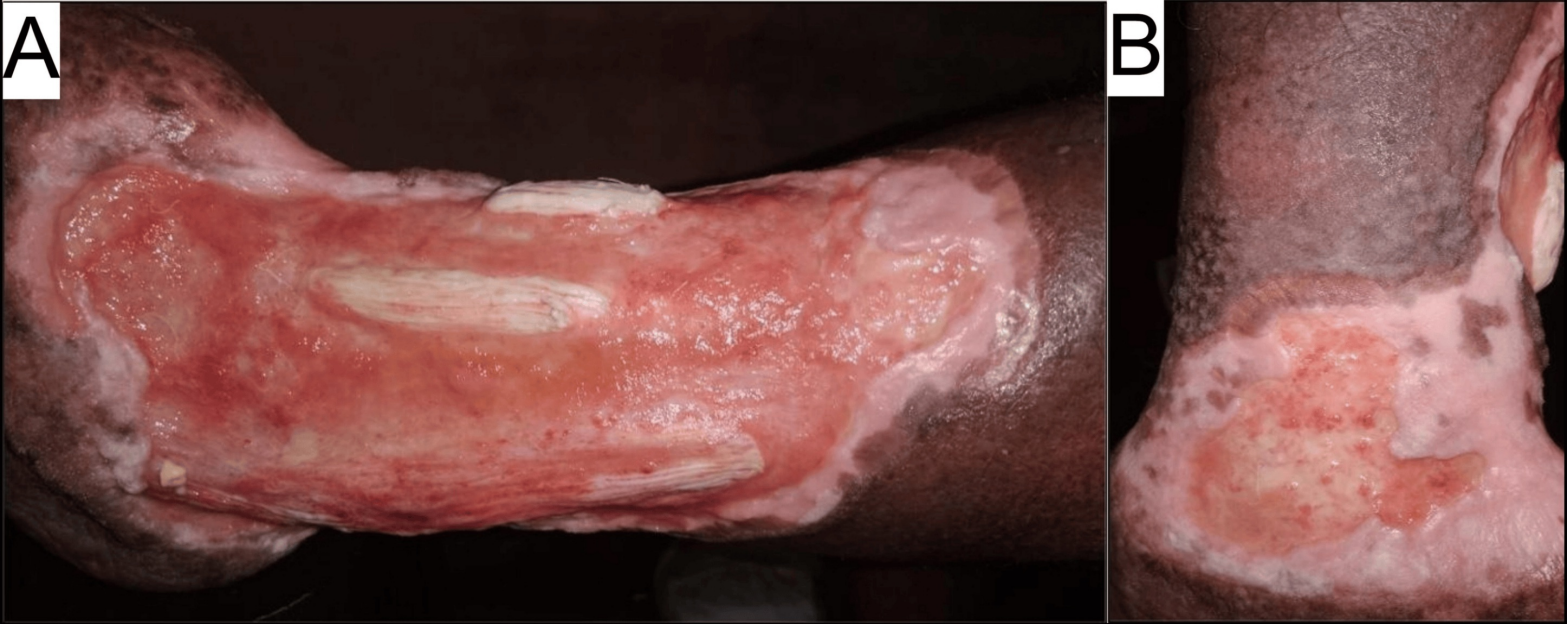
The pre skin graft ulcer with healthy granulation tissue and ulcer edges (A and B).

Initially, the wound was thoroughly irrigated with acetic acid, and a topical metronidazole dressing was applied. Regular topical metronidazole dressing was continued throughout the course of the ulcer as a prophylactic measure against infection. In addition, a seven-day course of oral co-amoxiclav 625 mg thrice daily was administered as a prophylactic measure, despite the wound culture being unremarkable for organism growth. The patient was also prescribed deflazacort 30 mg once a day, colchicine 0.5 mg twice a day, gabapentin 400 mg once a day, and amitriptyline 10 mg once a day to address inflammation and neuropathic pain, respectively. The dose of deflazacort was reduced by 6 mg every five days until 12 mg once a day, and this dose was kept as the maintenance dose. The previously mentioned dosages were continued for colchicine, gabapentin, and amitriptyline. After approximately eight months of our treatment, the inflammation subsequently subsided and showed healthy granulation tissue. Figures 3A, 3B show the ulcer with healthy granulation tissue and edges at eight months.

**Figure 4:**
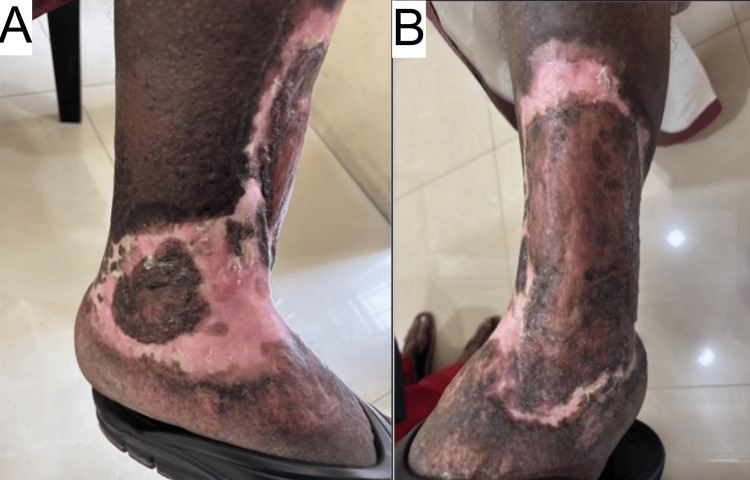
The images upon one-month follow-up post-surgical skin grafting (A and B).

He underwent a split-thickness skin graft surgery while maintaining the anti-inflammatory cover as an attempt to minimize graft rejection. The skin was harvested from the right thigh and was secured at the site using the stapler method. The procedure was completed in roughly an hour. Figures 4A, 4B show the one-month post-procedure results.

**Figure 5:**
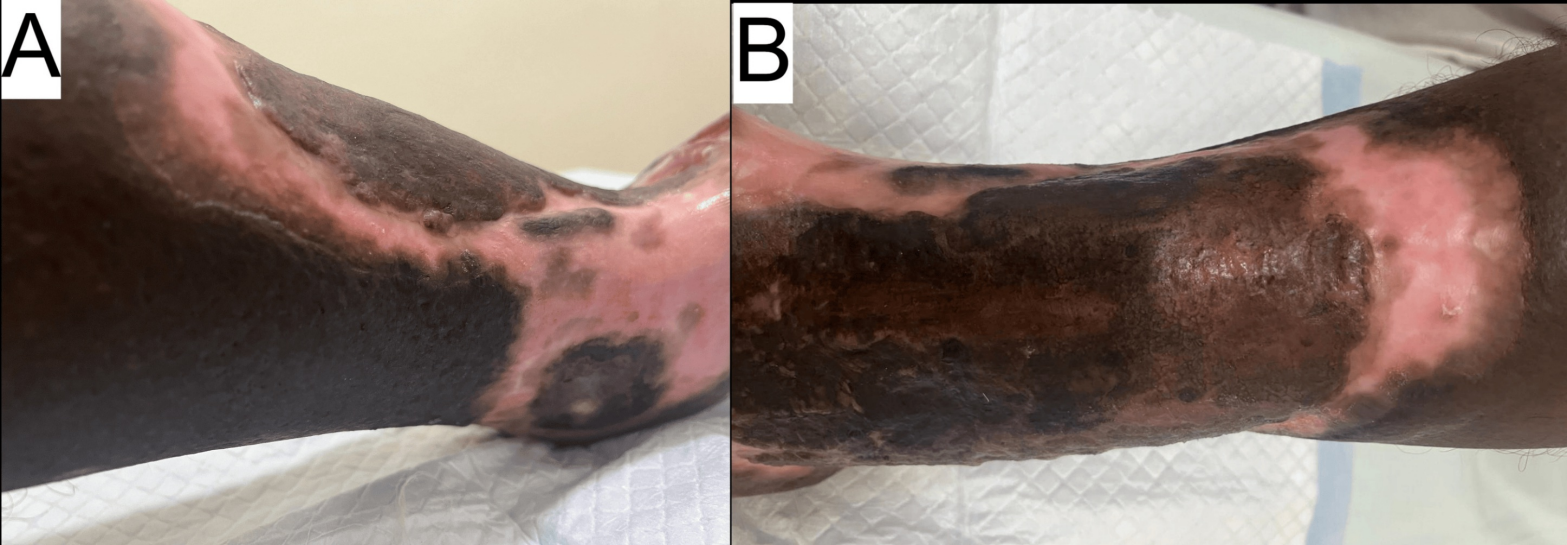
The one-year follow-up results (A and B).

The procedure was successful without any rejection. The patient was kept on deflazacort 12 mg once a day for four weeks after the procedure, was on 6 mg once a day between four and six weeks, and was off steroids beyond six weeks post-procedure. He did not develop any metabolic steroid-related side effects, such as Cushingoid features or hyperglycemia, during the period of steroid administration. Colchicine, amitriptyline, and gabapentin were also stopped after six weeks of the surgery. He continues monthly reviews at our hospital and remains ulcer-free even a year after the surgery. Figures 5A, 5B show the one-year follow-up pictures. The patient also reports improvement in his quality of life by being relieved of pain, the financial burden of regular wound dressing, and the psychological impact it had on him. He is able to attend his work more regularly, and he states his productivity has also improved, being ulcer-free.

## Discussion

This case illustrates the profound chronicity and therapeutic recalcitrance possible with snake bite-induced ulcers. Although chronic ulcers following snake bite are recognized sequelae, literature regarding management strategies for successful ulcer healing is sparse. Reconstructive surgery is a recognized strategy to treat lower extremity defects in snake bites [5]. However, there is inadequate evidence regarding its long-term results. A population-based study in Sri Lanka described a 33-year-old snake-bite pedal ulcer where there were multiple failed skin graft attempts [6].

Tissue-damaging effects of snake venom are recognized as the leading cause of morbidity in snake bite, leading to lifelong disabilities like permanent muscle loss, contractures, chronic kidney disease, and even chronic ulceration. The tissue-damaging venom components, such as phospholipases A_2_ (PLA_2_s) and snake venom metalloproteinases (SVMPs), induce a complex inflammatory response associated with the production and release of a large number of mediators and a prominent inflammatory infiltrate of neutrophils and macrophages [7].

Chronic ulcers are often found to be "stuck" in an inflammatory phase [8]. Shanmugam et al. demonstrated 23% prevalence of immune disease in chronic ulcers, significant rates of non-specific biopsy changes, and higher rates of surgical skin graft rejections in such patients [9]. Bosanquet et al. published a 10-year retrospective review showing that topical steroids combined with topical antibiotics and antifungal agents achieved faster wound healing in chronic ulcers [10]. Systemic corticosteroids, along with systemic immunosuppressants and biological drugs, can be used in inflammatory ulcers in addition to the local treatment strategies [11]. Colchicine via microtubule depolymerization inhibits cell proliferation, chemotaxis, and gene expression of neutrophils. It also has additional antimitotic, antifibrotic, anti-inflammatory, and immunosuppressive action with a good safety index. This makes colchicine a good candidate for various dermatological immune-mediated conditions, such as pyoderma gangrenosum, Behcet’s disease, vasculitis, recurrent aphthous stomatitis, dermatomyositis, scleroderma, etc. [12]. These factors, along with the fact that an ulcer duration of 30 years significantly affects quality of life, prompted us to start our patient on a dual corticosteroid and colchicine therapy. Our aim was to target the chronic inflammatory phase of the ulcer with anti-inflammatory agents, as described in our previously published case series [13]. Our patient had regular follow-ups to look out for secondary infections of the ulceration. After eight months, once sufficient healthy granulation tissue had developed, he underwent split-thickness skin grafting, which showed no graft rejection or re-ulceration. The patient has remained ulcer-free and healthy for more than a year since the surgery.

We hypothesize that the novel strategy to suppress chronic inflammation prior to the skin graft could have potentially played a role in helping skin grafting success. This strategy was employed keeping in mind that the patient had rejected surgical skin graft previously. Though we did not have a lot of documented data about this failed attempt, a few plausible explanations can be inferred from existing literature. Ulcers with autoimmune etiology have higher skin graft rejections compared to patients without autoimmune disease [14]. There could have been an underlying autoimmune component in this case, even though the biopsy did not reveal evidence of vasculitis as such. Confirmation of vasculitis in autoimmune ulcers, such as rheumatoid arthritis ulcers, is challenging, and only 50-55% of biopsies show vasculitis features. Clinical suspicion of autoimmune etiology should be considered in chronic refractory ulcers [14]. Skin grafting in larger non-healing ulcers (>100 cm²) is associated with a higher failure risk and longer healing [15].

Although we did not encounter any serious adverse events with our patient, the chronic administration of immunosuppressants, including steroids, should be employed with caution. The common side effects of steroid therapy include immunosuppression, susceptibility to systemic infection, hyperlipidemia, weight gain, cushingoid features, adrenal insufficiency, depression, and mood changes [16]. Diarrhea, nausea, vomiting, abdominal pain, cramping, and anorexia are commonly associated with colchicine use [17]. Hence, strict vigilance at all times should be maintained with their administration.

## Conclusions

This case sheds light on the possibility of managing chronic, refractory ulcers after snake-bite envenoming. The main strategy employed here was recognizing and targeting the underlying chronic inflammatory drive over an extended period before attempting surgical reconstruction. The combination of deflazacort and colchicine, alongside standard wound care, could have played a role in effectively suppressing inflammation, enabling successful surgical skin grafting where previous attempts had failed. However, larger studies are required to shed light on the pathophysiology of chronic non-healing ulcers in snake bite and successful management strategies.
